# Development of the anterior-posterior axis is a self-organizing process in the absence of maternal cues in the mouse embryo

**DOI:** 10.1038/cr.2015.104

**Published:** 2015-09-04

**Authors:** Ivan Bedzhov, Monika Bialecka, Agata Zielinska, Joanna Kosalka, Francesco Antonica, Amelia J Thompson, Kristian Franze, Magdalena Zernicka-Goetz

**Affiliations:** 1 Department of Physiology, Development and Neuroscience, University of Cambridge, Downing Street, Cambridge CB2 3DY, UK

## Dear Editor,

Establishment of the anterior-posterior (AP) axis in the mouse embryo is crucial for proper induction of the germ layers. Despite this importance, it is unclear whether specification of the AP axis is an autonomous process or if it requires an embryonic-maternal interaction at the time of implantation. By embryonic day 5.5 (E5.5), the combined activities of Nodal, GDF3 and the Cripto co-receptor generate a proximal-distal gradient that establishes a signaling center at the distal tip of the embryo ― the distal visceral endoderm (DVE). This proximo-distal asymmetry is transformed into the AP axis as the DVE cells migrate. DVE cells express the Nodal antagonists, Lefty1 and Cer1, which specify the anterior identity of the underlying epiblast^1^. The expression of these key DVE markers is already triggered before implantation^2,3,4^. Cells that express Lefty1 and Cer1 in the late blastocyst contribute to DVE formation, together with cells that acquire *de novo* Cer1 and Lefty1 expression after implantation^3,4,5^. This pre-implantation expression of the Nodal antagonists led to the suggestion that the DVE, and therefore the AP axis, arise independently of any interaction between mother and embryo.

However, recently an alternative mechanism was proposed. By growing early post-implantation embryos in gels of different agarose concentrations Hiramatsu *et al*.^6^ found that DVE is established only in stiffer gels, concluding that *in vivo* the maternal tissues should exert similar mechanical forces to enable DVE formation. Moreover, this biomechanical stress was reported to result in the transmigration of epiblast cells into the visceral endoderm (VE), which were suggested to generate the DVE at the distal tip. Therefore, two fundamentally different concepts about the mother's role in AP axis specification are currently considered.

To resolve whether establishment of the AP axis depends on mother-embryo interactions, or if it is an embryo-autonomous process, we performed a series of embryo culture and lineage tracing experiments. We used a Cer1-GFP line, previously established as a faithful DVE reporter^6,7^, to monitor DVE formation and migration directly in living embryos.

**Figure 1:**
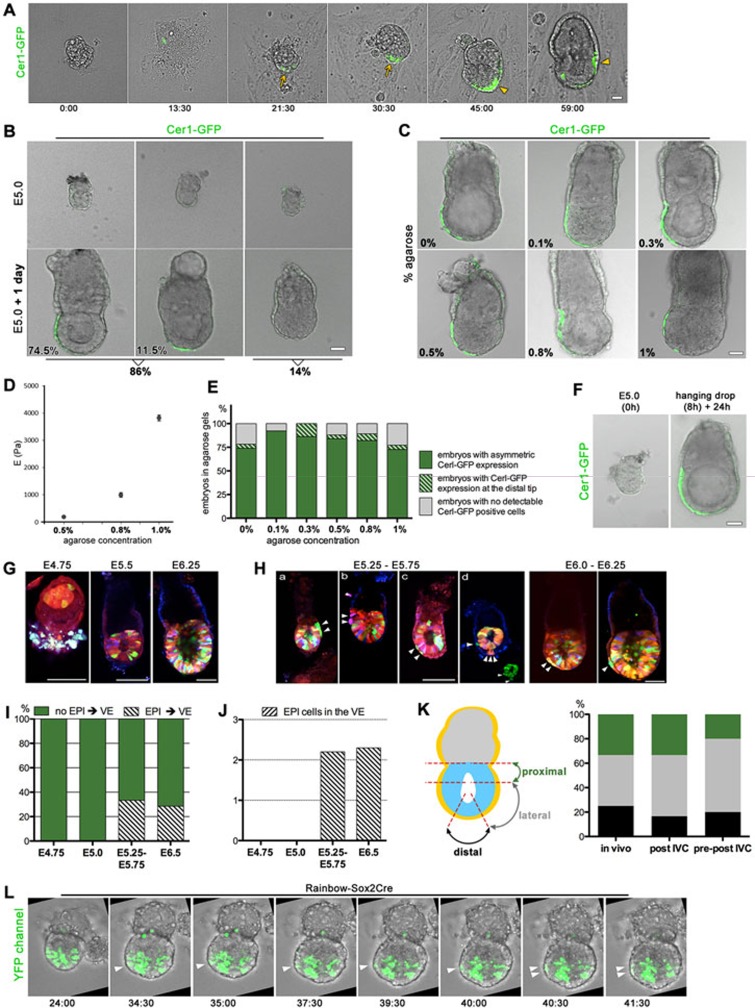
Mouse embryos autonomously establish the AP axis. **(A)** Still images of time-lapse recording of egg cylinder formation *in vitro*. Cer1-GFP domain positions at the distal tip (arrows) and migrates proximally (arrowheads). **(B)** E5.0 embryos (*n* = 59) individually cultured for 24-30 h and classified according to the Cer1-GFP expression pattern. **(C)** Representative pictures of embryos expressing Cer1-GFP unilaterally after culture in gels with various agarose concentrations. **(D)** Plot of average median Young's moduli with increasing agarose concentration: 0.5%, 0.8% and 1% w/v (*n* = 3 gel replicates, 10-15 measurements per replicate); 0.1% and 0.3%, not measurable. Error bars denote SEM. **(E)** Quantification of Cer1-GFP expression pattern in embryos developing in agarose gels. **(F)** Representative pictures of E5.0 embryos (*n* = 40) developing in hanging drops for 8 h followed by an additional 24 h culture in four-well plates. **(G)** Color conversion of epiblast cells in E4.75-E6.25 Rainbow-Sox2Cre embryos. **(H)** Representative pictures of labelled epiblast cells in the VE: (a, b) proximal position; (c) lateral; (d) lateral and distal. Note that in d there are cells at different positions and of different colors in VE (for clarity the inset shows YFP only). **(I)** Proportion of E4.75-E6.5 embryos with labelled cells in the VE (embryos examined: E4.75, *n* = 7; E5.0, *n* = 6; E5.25-E5.75, *n* = 33; E6.5, *n* = 16); EPI→VE denotes embryos with epiblast-derived cells transmigrated to VE. **(J)** Average number of transmigrated cells in E4.75-E6.5 embryos. **(K)** Schematic representation of the areas in which transmigrated epiblast cells were observed and quantification of the number of cells at particular positions in embryos. *In vivo*, embryos were recovered at E5.25-E5.75; post-IVC, embryos were recovered at the post-implantation stage and filmed *in vitro*; pre-post IVC, embryos were recovered at the pre-implantation stage and filmed *in vitro*. **(L)** Still frames from movies showing epiblast-to-VE transmigration in a Rainbow-Sox2/Cre embryo recovered at the blastocyst stage and cultured *in vitro*. Scale bars: **A-C** and **F**, 50 μm; **G** and **H**, 100 μm.

First, we recovered embryos before implantation, at E3.5, and monitored their development and DVE formation through implantation stages *in vitro* on plastic plates, as we previously described^5,8,9^. Cer1-expressing cells became detectable after 1-1.5 day of culture and as development progressed they consolidated at the distal tip to establish the DVE (Supplementary information, Figure S1A, S1B and Movie S1). To capture the dynamics of DVE formation, we imaged pre- to post-implantation morphogenesis by time-lapse microscopy (Figure 1A and Supplementary information, Movie S2). We analyzed 66 embryos and found that 87.8% established a Cer1-expression domain during the *in vitro* culture: in 58.3% it was positioned asymmetrically, and in 29.5% it remained located distally. Only in 12.2% of the embryos a Cer1-expression domain was not detectable (Supplementary information, Figure S1C and S1D). The migratory Cer1-expressing cells assembled into string-like configurations, extending in a distal-to-proximal direction (Supplementary information, Figure S1E). We analyzed 39 divisions of Cer1-expressing cells in seven *in vitro* developing embryos, of which 69.9% were parallel to the direction of migration (Supplementary information, Figure S1E-S1G), suggesting that proliferating DVE cells become organized due to their pattern of divisions and migratory properties.

Next, we asked whether peri-implantation embryos (E5.0) could also establish the DVE in the absence of interactions with the mother. We analyzed the development of 59 E5.0 embryos and found that after 1 day of culture, 86% of them established a Cer1-expression domain: in 74.5% it was positioned asymmetrically and in 11.5%, it was located distally. Only 14% of the embryos lacked detectable Cer1 expression (Figure 1B, Supplementary information, Figure S2A-S2C, S2I, and S2J). Time-lapse microscopy confirmed the emergence of Cer1-expressing cells that extruded filopodia-like protrusions during their migration (Supplementary information, Figure S2K and Movie S3). As controls, we cultured 66 E5.5 embryos that had already established the DVE *in utero* by that stage in 87.9% of cases (Supplementary information, Figure S2D-S2F). Upon culture, the Cer1-expression domain moved asymmetrically in 81.3% of embryos (Supplementary information, Figure S2G, S2H and S2J). These observations were confirmed using another DVE reporter Lefty1(mVenus)^2^. Both E5.0 and E5.5 Lefty1(mVenus) embryos established asymmetric Lefty1 expression during 24 h of culture, without the requirement of external biomechanical pressure (Supplementary information, Figure S3A-S3C).

To investigate whether DVE establishment and migration are influenced by substrate stiffness, as recently proposed^6^, we followed the methodology reported by Hiramatsu *et al*. and cultured 146 E5.0 embryos in gels of various agarose concentrations: 0% (*n* = 23 embryos), 0.1% (*n* = 26), 0.3% (*n* = 22), 0.5% (*n* = 25), 0.8% (*n* = 28) and 1% agarose (*n* = 22; Figure 1C). Atomic force microscopy indentation measurements of the gels yielded Young's moduli of 3820 ± 100 Pa (mean ± SEM) for 1% w/v agarose, 990 ± 80 Pa for 0.8% w/v agarose, and 190 ± 10 Pa for 0.5% w/v agarose (Figure 1D). We found that in all agarose concentrations embryos were able to establish a Cer1-expression domain that migrated asymmetrically (Figure 1C and 1E). Moreover, embryos cultured under all of these conditions grew and elongated to similar extents (Supplementary information, Figure S4A).

To exclude any possibility that interaction of the embryo with the substrate provides a mechanical stimulus leading to DVE formation, we cultured embryos free-floating in hanging drops of medium. We first cultured 40 E5.0 embryos, from which the majority (75.8%, *n* = 40) established a Cer1-expression domain and in 60.7% of cases this domain translocated anteriorly (Figure 1F, Supplementary information, Figure S4B and S4C). To determine whether the DVE is established in free-floating embryos from an even earlier stage, we cultured blastocysts in hanging drops. Again the majority of embryos (77.2%, *n* = 42) developed into egg cylinders that established a Cer1-expression domain and in 54.3% of embryos this domain translocated anteriorly (Supplementary information, Figure S4D-S4E).

Together, these experiments demonstrate that the DVE can form and migrate without a requirement of external spatial restriction on the embryo. These results are consistent in all experimental conditions irrespective of whether the embryos developed on a plastic substrate, in gels of varying stiffness or in hanging drops.

It has also been proposed that physical constriction of the embryo leads to transmigration of epiblast cells into VE to generate the DVE^6^. Since our results strongly indicate that DVE can form without any need of physical constriction, we next sought to determine whether the epiblast transmigration is essential for DVE formation. To this end, we used Rainbow transgenic mice^10^ in combination with mice carrying a Sox2Cre transgene^11^ to follow individual genetically labelled epiblast cells.

The first labelled epiblast cells in a color other than the default red were detected at E4.75 (*n* = 7; Figure 1G). While analyzing Rainbow-Sox2Cre embryos, we found that when the proamniotic cavity starts expansion (E5.25), a small proportion of embryos had cells of epiblast origin in the VE. We analyzed 33 embryos recovered at E5.25-E5.75 and found that 33% had epiblast-originating cells in VE (Figure 1H and 1I). On average, two cells of epiblast-origin were present in the VE per embryo (Figure 1J). Such cells in an embryo were the same color and usually in close proximity to each other, suggesting that they were sister cells. In two cases two non-adjacent cells of different colors were found in the VE (Figure 1Hd), raising the possibility that the epiblast can contribute to VE on more than one occasion in the same embryo. The position of epiblast-originating cells was not skewed to a particular site in the embryo: in three cases, epiblast-originating cells in the VE were in close proximity to the embryonic-extra-embryonic junction; in five cases, in the lateral part of the VE; and in four cases, at the distal tip (Figure 1K (*in vivo*)). A similar situation was observed at E6.5, where 4 out of 16 embryos had epiblast-originating cells in VE (29%, Figure 1I). As with E5.25-E5.75 embryos, E6.5 embryos had an average of two epiblast-originating cells in the VE that were of the same color and always in the lateral part of the VE (Figure 1J). The presence of epiblast-originating cells in the VE suggests that epiblast cells transmigrate as the embryo develops. To test that, we filmed the *in vitro* development of Rainbow-Sox2Cre embryos recovered at E5.25 or E5.75 by time-lapse microscopy. In 14 filmed embryos, we found 6 in which epiblast cells transmigrated into the VE at random positions (Figure 1K (post-IVC), Supplementary information, Figure S5A and S5B).

Finally, to determine whether the epiblast-to-VE transmigration could occur in the absence of any physical influence of the maternal environment, we filmed the *in vitro* development of blastocysts into egg cylinders (*n* = 14). As the pro-amniotic cavity started expansion, a single epiblast cell transmigrated into the VE in half of observed embryos (Figure 1L, Supplementary information, Figure S5A and Movie S4). These transmigrated cells became embedded at different positions in the VE or excluded from the embryo and in none of the cases contributed to the DVE (Figure 1K (pre-post-IVC) and 1L).

These results demonstrate that the epiblast-to-VE transmigration does take place, which is in agreement with the recent report^6^. However, the results we present here indicate that the epiblast-to-VE transmigration occurs not just at the distal tip, but in random positions along the egg cylinder, and that it is not required for DVE formation.

In a series of embryo culture experiments in different environmental conditions we show here that the AP axis in the mouse embryo can be specified in the absence of embryo-maternal interaction and without any external constraint. These findings contradict the suggestion that maternal tissues are essential to exert mechanical forces that enable DVE formation^6^. In the previous study, the authors report that it is necessary to encase embryos in an agarose gel in order to induce DVE formation *in vitro*. Moreover, gels containing less than 0.5% agarose were described as unable to support embryo elongation and DVE formation, contrary to our observations. How might the differences between these results arise? One possibility is the method of embryo culture. Whereas medium containing rat serum was used by Hiramatsu *et al*., here we culture embryos in a serum-free medium. Initially we attempted a similar embryo culture approach reported by Hiramatsu *et al*.; however we were unable to obtain a batch of rat serum of sufficient quality to support normal embryo growth (data not shown). To overcome this obstacle, we used the chemically defined IVC2 medium that we have recently established, which supports embryonic development beyond the blastocyst stage^8,9^. It is possible that the defined medium has an advantage over the serum-containing media as it excludes variability between batches of serum and the possibility of supplying growth factors and morphogens found in serum that might influence the outcome of the culture. Therefore, our results indicate that if satisfactory culture conditions are provided, the DVE can be specified outside the maternal environment in the absence of external pressure. Together, our results suggest that the processes by which the DVE forms and migrates to set up the AP axis are embryo-autonomous, illustrating the self-organizational properties of the mouse embryo.

## Supplementary Information

Supplementary information, Figure S1 DVE specification during blastocyst-to-egg cylinder transition *in vitro*.

Supplementary information, Movie S1 Time-lapse movie showing the appearance of Cer1-GFP expressing cells during the *in vitro* culture of E3.5 Cer1-GFP embryo. Related to Figure 1 and Supplementary Figure S1.

Supplementary information, Movie S2 Time-lapse movie of DVE formation and migration in Cer1-GFP embryo developing from blastocyst stage *in vitro*. Related to Figure 1 and Supplementary Figure S1.

Supplementary information, Movie S3 Time-lapse movie of DVE formation and migration in Cer1-GFP embryo developing in the absence of external mechanical pressure from E5.0. Related to Figure 1 and Supplementary Figure S2.

Supplementary information, Movie S4 Time-lapse movie (3D projection) of embryo cultured from E4.75 *in vitro* with frame interval 30min. YFP channel only (left panel) and YFP/bright filed merge (right panel). Arrowheads point to epiblast cell that transmigrates to VE. White line denotes the EPI-VE border. Related to Figure 1 and Supplementary Figure S4.
