# Supplementary material for: Development of the anterior-posterior axis is a self-organizing process in the absence of maternal cues in the mouse embryo
Source: Cell Res. 2015 Sep 4;25(12):1368–71. doi: 10.1038/cr.2015.104 (PMC4670986; doi:10.1038/cr.2015.104)
Supplement: Supplementary information, Figure S1 — DVE specification during blastocyst-to-egg cylinder transition in vitro. [file cr2015104x1.pdf]

Supplementary Figure **S1**

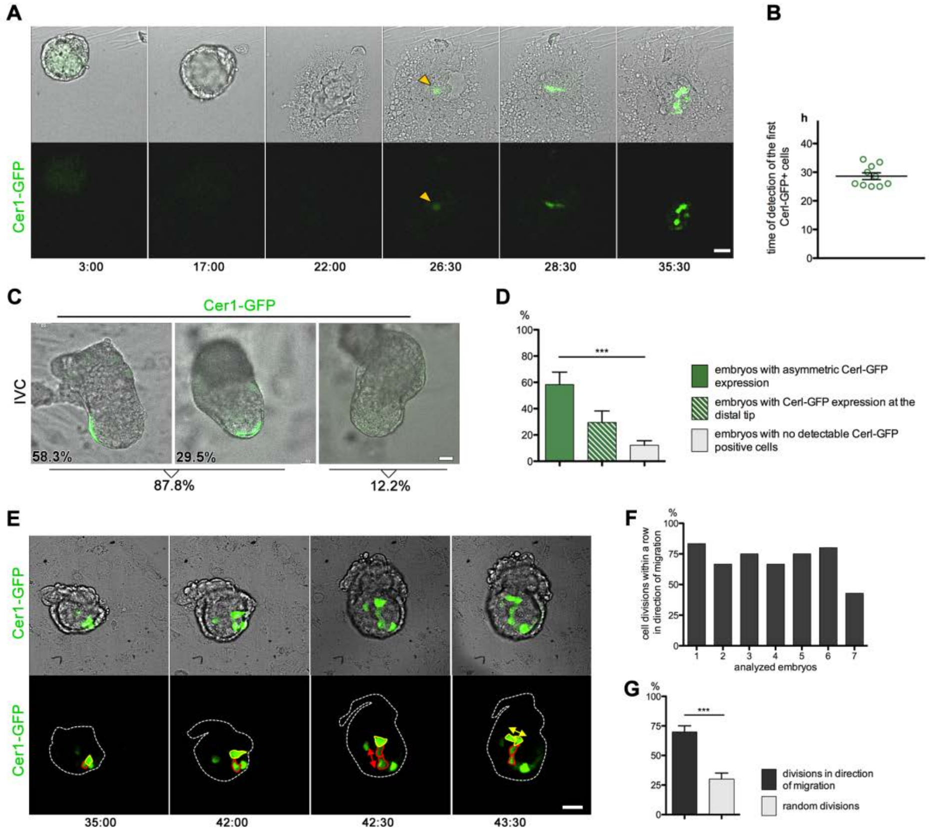

Supplementary information, Figure S1. DVE specification during blastocyst-to-egg cylinder transition *in vitro.* (A) Still images of time-lapse recording of blastocyst attaching to the substrate with first Cer1-GFP cells appearing (green), indicated with an arrowhead. (B) Time of detection of the first Cer1-GFP expressing cells (n=10 embryos). (C) Representative pictures of embryos that formed egg cylinders with Cer1-expressing domain localized: asymmetrically (left), at the distal tip (middle) and with no detectable Cer1-GFP expression (right). (D) 58.3% of the blastocysts that developed into egg cylinders *in vitro* have asymmetric Cer1-GFP expression, 29.5% of embryos show Cer1-GFP expression at the distal tip in and in 12.2% of embryos Cer1-GFP expression is

not detectable (n=66 embryos; more than 3 independent experiments, ***p <0.05, t test). (E) Still images of time-lapse recording of migrating Cer1-GFP cells. Cell divisions are indicated with double arrowheads. (F) Cell divisions within a row in direction of migration in 7 *in vitro* developed egg cylinders (n=39 cells divisions). (G) Divisions in direction of cell migration (69.9%) versus divisions that appeared to be randomly oriented (n=7 embryos, 39 cell divisions, ***p <0.05, t test). Error bars represent SEM. Scale bars = 50μm. Related to Figure 1. See also Movie S1 and Movie S2.

Supplementary Figure **S2**

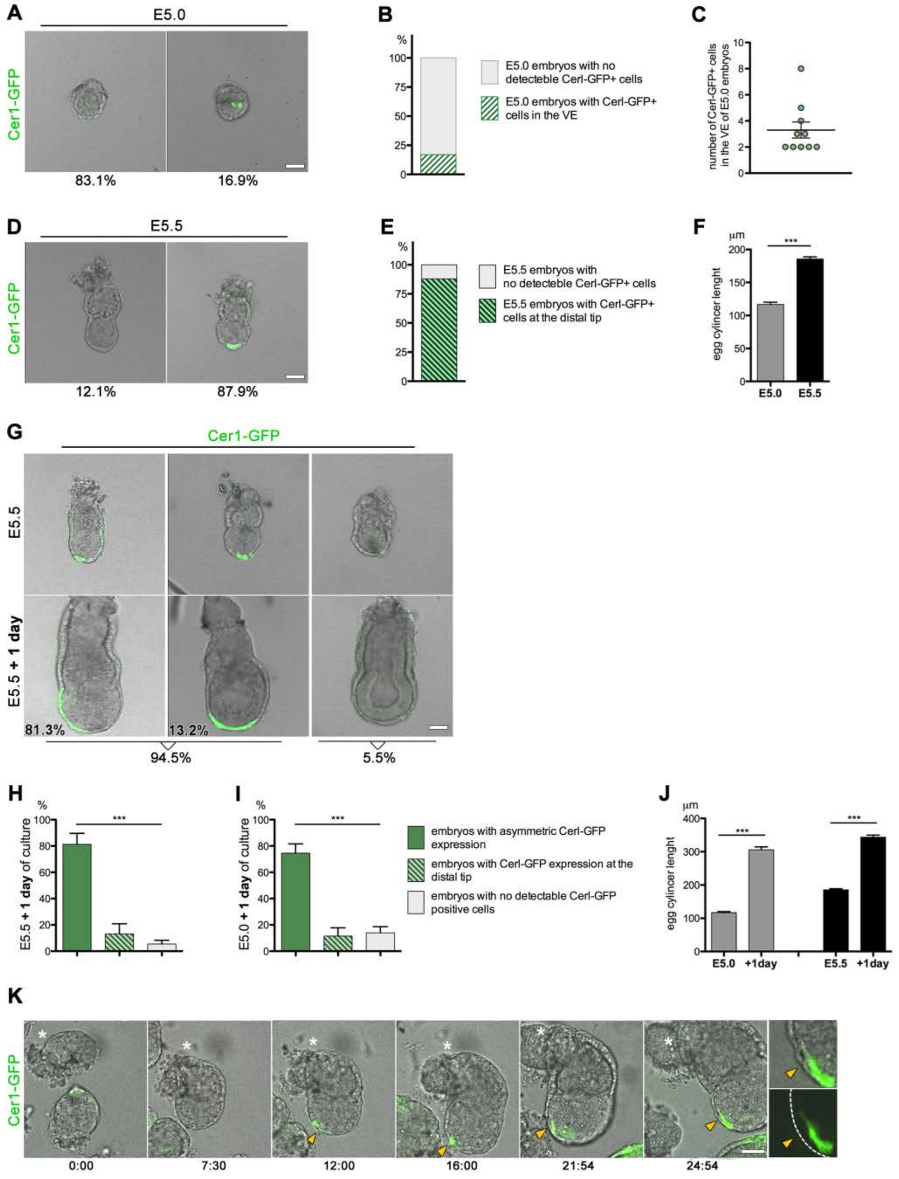

Supplementary information, Figure S2. Establishment of DVE in the absence of external mechanical cues. (A) E5.0 embryos classified according to the presence Cer1-GFP expression domain in VE (n=59 embryos). (B) Cer1-GFP expressing cells were found in 16.9% of the E5.0 embryos and in 83.1% Cer1-GFP expression was not detected

(n=59 embryos, more than 3 independent experiments). (C) Number of Cer1-GFP expressing cells in the VE of E5.0 embryos (n=10 embryos, more than 3 independent experiments). (D) E5.5 embryos classified according to the Cer1-GFP expression (n=66 embryos). (E) Cer1-GFP expressing cells were positioned at the distal tip in 87.9% of the E5.5 embryos and in 12.1% Cer1-GFP expression was not detected (n=66, more than 3 independent experiments). (F) Average length of E5.0 (n=59 embryos) versus E5.5 (n=66) embryos (***p <0.05, t test). (G) E5.5 embryos individually cultured for a day (24-30h) in single wells of 96 well plates containing IVC2 medium and classified according to the Cer1-GFP expression pattern (n=66, more than 3 independent experiments). (H) After one day of culture 81.3% of the E5.5 embryos showed asymmetric Cer1-GFP expression; 13.2%, expression at the distal tip and in 5.5% Cer1-GFP signal was not detected (n=66, more than 3 independent experiments, ***p <0.05). (I) 74.5% of the E5.0 embryos showed asymmetric Cer1-GFP expression after one day of culture; 11.5%, expression at the distal tip and in 14% Cer1-GFP signal was not detected (n=59, more than 3 independent experiments, ***p <0.05). (J) Average length of E5.0 (n=59 embryos) and E5.5 (n=66) embryos at the time of recovery and after one day of culture (***p <0.05). (K) Still images of time-lapse recording of E5.0 embryos cultured without mechanical restriction, establishing Cer1-GFP expression (arrowheads) at the distal tip, followed by proximal migration at one side of the elongating egg cylinder (n=11 embryos). Error bars represent SEM. Scale bars = 50μm. Related to Figure 1. See also Movie S3.

Supplementary Figure **S3**

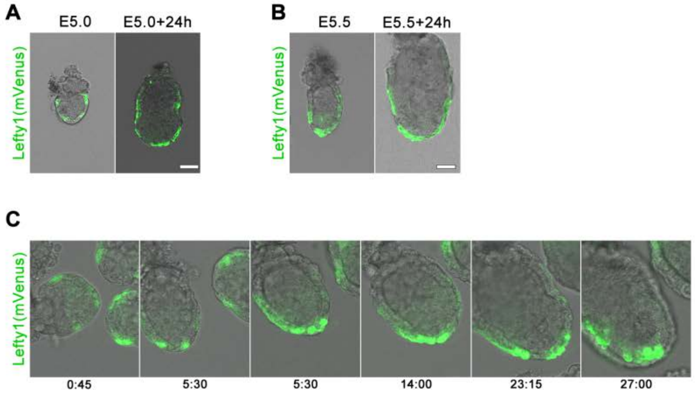

Supplementary information, Figure S3. Embryo autonomous establishment and migration of DVE in Lefty1(mVenus) embryos. Representative pictures of (A) E5.0 embryos (n=8 embryos) and (B) E5.5 embryos (n=33 embryos) cultured in vitro for 24h. (C) Still images of time-lapse recording of E5.0 embryo cultured without an external mechanical constrain, establishing Lefty1(mVenus) positive domain at one side of the elongating egg cylinder. Scale bars = 50μm. Related to Figure 1.

Supplementary Figure **S4**

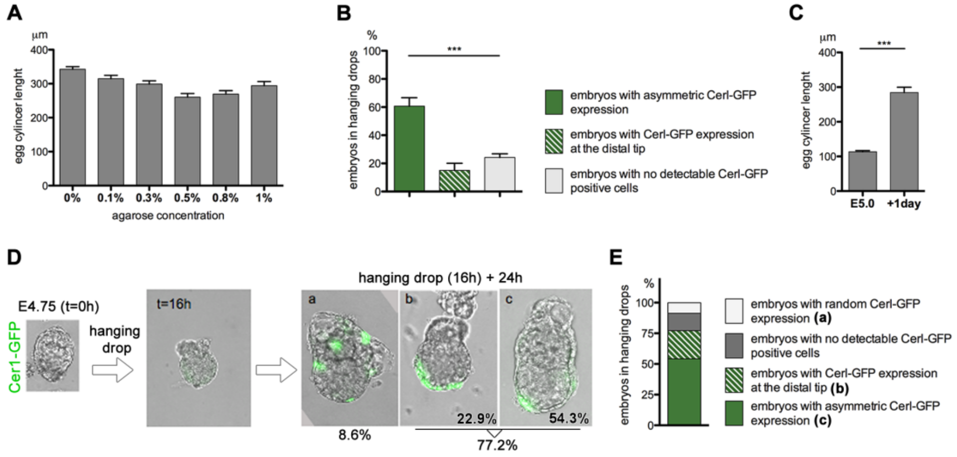

Supplementary information, Figure S4. Embryo-autonomous establishment of the DVE. (A) Average length of egg cylinders cultured in 0% to 1% agarose containing gels. (B) After 8h hanging drop plus 24h additional culture 60.7% of the E5.0 embryos showed asymmetric Cer1-GFP expression; 15.1%, expression at the distal tip and in 24.2% Cer1-GFP signal was not detected (n=40 embryos, more than 3 independent experiments, ***p <0.05). (C) Average length of the E5.0 egg cylinders (n=40 embryos) at the time of recovery and at the end of the culture (***p <0.05). (D) Representative pictures of E4.5 embryos that establish egg cylinders (n=35) as they were grown individually in hanging drops of 1:1 ratio of IVC1:IVC2 media for 16h and then for additional 24h in a 4-well plates. (E) After 16h hanging drop plus 24h additional culture 54.3% of the egg cylinders showed asymmetric Cer1-GFP expression; 22.9%, expression at the distal tip; 8.6%, random Cer1-GFP expression and in 14.2% Cer1-GFP signal was not detected (n=35 embryos, more than 3 independent experiments). Error bars represent SEM. Scale bars = 50μm. Related to Figure 1.

Supplementary Figure **S5**

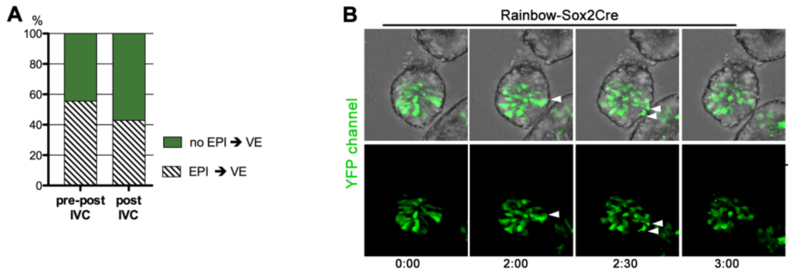

Supplementary information, Figure S5. Transmigration of epiblast cells to the VE. (A**)** Graph representing the amount of cultured embryos with and without epiblast cells transmigrating to the VE. Pre-post IVC: embryos cultured from E4.75 that reached the egg cylinder stage (n=9); post IVC: cultured early post-implantation embryos (n=14). (B) Still frames from movies showing transmigration of epiblast cell to VE in a Rainbow-Sox2Cre post-implantation embryo. See also Movie S4. Related to Figure 1.

Supplementary information, Data S1 Materials and Methods

Blastocyst to egg cylinder *in vitro* culture and time-lapse imaging of Cer1-GFP embryos

Embryos were recovered from spontaneously ovulated F1 females mated with Cer1-GFP males[1]. Culture medium and conditions were as previously described [2, 3]. Blastocysts were cultured in IVC1 medium (Cell guidance systems - M11-25), (IVC1 - Advanced DMEM/F12 (Gibco) containing 20% FCS (Biosera) and supplemented with 2 mM L-glutamine (Gibco), 1 mM Sodium pyruvate (Gibco), Penicillin (25 units/ml) / Streptomycin (25 µg/ml) (Gibco,), 1x ITS-X (Invitrogen), 8 nM β-estradiol (Sigma), 200 ng/ml Progesterone (Sigma) and 25 µM N-acetyl-L-cysteine (Sigma)). After 48h the IVC1 medium was exchanged with serum-free IVC2 medium (Cell guidance systems - M12-25), (IVC2 - Advanced DMEM/F12 (Gibco) containing 30% KSR (KnockOut Serum Replacement, Gibco) and supplemented with 2 mM L-glutamine (Gibco), 1 mM Sodium pyruvate (Gibco), Penicillin (25 units/ml) / Streptomycin (25 µg/ml) (Gibco), 1x ITS-X (Invitrogen), 8 nM β-estradiol (Sigma), 200 ng/ml Progesterone (Sigma) and 25 µM N-acetyl-L-cysteine (Sigma)). Time-lapse microscopy was performed using a spinning-disc microscope (Intelligent Imaging Innovations) with acquisition interval of 15-30 min, every 8 µm z-planes, at 37°C, in 5% CO_2. The images were analyzed using Slidebook 5.0 (Intelligent Imaging Innovations) and Imaris 7.5 (Bitplane) software.

Culture of early post-implantation embryos

After recovery, the Reichert's membrane of E5.0 and E5.5 embryos was dissected out and Cer1-GFP expression assessed using a Leica SP5 confocal microscope. The embryos were cultured individually in 96 well plates filled with IVC2 medium for 1 day (24-30h). Cer1-GFP pattern was re-examined at the end of the culture.

Hanging drop culture

The mural trophectoderm of E4.75 blastocysts was dissected out as previously described [2]. The embryos were individually cultured in hanging drops containing 50% IVC1 and 50% IVC2 media. After 16h of hanging drop culture the embryos were transferred in 4-well plates filled with IVC2 media for additional 24h of culture. E5.0 embryos were cultured individually in 30 μl hanging drops of IVC2 media for 8h and then transferred in 4-well plates for additional 24h.

Atomic force microscopy

Agarose gels (1%, 0.8% or 0.5% w/v) were fabricated in plastic Petri dishes (TPP, Switzerland) to a thickness of 3-4 mm. Tipless silicon cantilevers were calibrated using the thermal noise method [4] and cantilevers with spring constants between 0.07 and 0.08 N/m selected. Monodisperse spherical polystyrene beads (37.28 ±0.34 μm diameter; microParticles GmbH, Berlin, Germany) were glued to the cantilever ends as probes. Prepared cantilevers were mounted on a JPK CellHesion-200 AFM head (JPK Instruments AG, Berlin, Germany), which was set up on the stage of an inverted optical microscope (Axio ObserverA1, Zeiss, UK) above the gel sample. Force-distance curves (maximum indentation force 10-30 nN; approach speed 8 μm/s) were collected at 10-15 randomly selected points on 3 different gels per agarose concentration. Measurements were performed at a constant temperature of 26°C, maintained using a PetriDishHeater (JPK Instruments AG, Berlin, Germany). The Young's Modulus, a measure of elastic stiffness, was calculated using the built-in JPKSPM Data Processing software (JPK Instruments AG, Berlin, Germany). Raw AFM data was fitted to the Hertz model [5]:

$$F = \frac{4}{3}\frac{E}{1-\nu^2} r^{1/2}\delta^{3/2}$$

with applied force F, Young's modulus E, Poisson's ratio ν, indenter radius r, and

indentation depth δ, assuming $\nu = 0.5$ [6].

Lineage tracing and time-lapse imaging using Rainbow/Sox2Cre embryos

Embryos used in lineage tracing experiments were recovered from Rainbow line females [7] mated with males from a Sox2Cre transgenic line [8]. All embryos were scanned alive using a Leica SP5 confocal microscope at excitation wavelengths 434 for CFP, 514 for eYFP, 563 for dTomato and for the purpose of analysis, blue, green and red were assigned to CFP, eYFP and dTomato, respectively. Embryos were cultured for 24-30h in IVC2 medium and filmed using a spinning disc microscope (Intelligent Imaging Innovations) with acquisition interval of 30min.

References for Supplementary Materials and Methods

1 Mesnard D, Filipe M, Belo JA, Zernicka-Goetz M. The Anterior-Posterior Axis Emerges Respecting the Morphology of the Mouse Embryo that Changes and Aligns with the Uterus before Gastrulation. *Current Biology* 2004; 14:184-196.

2 Bedzhov I, Leung CY, Bialecka M, Zernicka-Goetz M. In vitro culture of mouse blastocysts beyond the implantation stages. *Nature protocols* 2014; 9:2732-2739.

3 Bedzhov I, Zernicka-Goetz M. Self-Organizing Properties of Mouse Pluripotent Cells Initiate Morphogenesis upon Implantation. *Cell* 2014; 156:1032-1044.

4 Hutter JL, Bechhoefer J. Calibration of atomic- force microscope tips. *Review of Scientific Instruments* 1993; 64:1868-1873.

5 Hertz H. Ueber die Berührung fester elastischer Körper. Journal für die reine und angewandte Mathematik (Crelle's Journal) 1882:156.

6 Normand V, Lootens DL, Amici E, Plucknett KP, Aymard P. New insight into agarose gel mechanical properties. *Biomacromolecules* 2000; 1:730-738.

7 Tabansky I, Lenarcic A, Draft RW *et al.* Developmental bias in cleavage-stage mouse

blastomeres. *Current biology : CB* 2013; 23:21-31.

8 Hayashi S, Lewis P, Pevny L, McMahon AP. Efficient gene modulation in mouse epiblast using a Sox2Cre transgenic mouse strain. *Mechanisms of Development* 2002; 119, Supplement:S97-S101.
